# MR-FuSN: A Multi-Resolution Selective Fusion Approach for Bearing Fault Diagnosis

**DOI:** 10.3390/s25041134

**Published:** 2025-02-13

**Authors:** Lin Sha, Shikai Tang, Min Wang, Sibo Qiao, Shihang Yu, Weixia Liu, Jiaqi Li

**Affiliations:** 1 School of Control Science and Engineering, Tiangong University, No. 399 Binshui West Road, Tianjin 300387, China; 2 School of Life Sciences, Tiangong University, No. 399 Binshui West Road, Tianjin 300387, China; 3 The School of Software, Tiangong University, No. 399 Binshui West Road, Tianjin 300387, China; 4 The School of Information and Control Engineering, Qingdao University of Technology, No. 777, Jialingjiang East Road, Qingdao 266520, China

**Keywords:** multi-resolution, selective networks, deep learning, fault diagnosis, vibration signal

## Abstract

Vibration signals serve as the primary data source for bearing fault diagnosis. However, when collected in complex industrial environments, these signals are often contaminated by noise interference, posing significant challenges to fault feature extraction and diagnostic accuracy. To address these issues, this paper proposes a novel bearing fault diagnosis network architecture: the Multi-Resolution Fusion Selection Network (MR-FuSN). The MR-FuSN effectively extracts domain-invariant features from input data through multi-resolution feature extraction and incorporates an adaptive kernel convolution strategy, thereby enhancing its robustness against environmental noise. Experimental results demonstrate that the MR-FuSN achieves outstanding performance in noisy environments with signal-to-noise ratios (SNRs) ranging from −5 dB to 10 dB, particularly attaining a diagnostic accuracy of 99.97% under 0 dB conditions. This study provides technical support for practical fault diagnosis applications.

## 1. Introduction

As indispensable components in rotating machinery, bearings are crucial for ensuring the stability and reliability of an entire mechanical system. These components support and guide rotating elements, reduce friction, and transmit loads, which are essential for the smooth operation of machinery [1]. Owing to their continuous operation under various loads and conditions, bearings are prone to failure in mechanical equipment [2]. These failures are often caused by excessive loads, inadequate lubrication, or material fatigue [3]. For instance, excessive load can lead to deformation or cracking, insufficient lubrication can increase friction and wear, and material fatigue may cause gradual degradation of bearing materials. Unexpected bearing failures can result in significant equipment damage and pose safety risks to operators [4], potentially leading to costly downtimes and hazardous situations [5].

In the initial stages of bearing fault diagnosis, traditional signal analysis techniques such as wavelet transform [6], Fourier analysis [7], and empirical mode decomposition [8] have been widely used because of their effectiveness in decomposing complex vibration signals into simpler components for analysis. These methods aid in identifying characteristic frequencies and patterns associated with specific types of faults. For example, wavelet transform, which can analyze signals at multiple scales, has proven to be particularly effective for detecting transient features in vibration signals. Fourier analysis, which is known for its frequency domain representation, can identify periodic components in the signal. Empirical mode decomposition can extract intrinsic mode functions from nonlinear and nonstationary signals, helping detect subtle fault features. For instance, He et al. [9] developed an innovative method combining wavelet packet transform with convolutional neural networks and optimized it using simulated annealing to improve diagnostic accuracy. This approach leverages the strengths of signal processing and deep learning to enhance fault detection capabilities. Similarly, Sun et al. [10] improved the wavelet transform technique by adjusting the modal maximum of vibration features to highlight defect-related characteristics, thereby enhancing the sensitivity of the diagnostic process. Qin et al. [11] proposed a novel m-band flexible wavelet transform specifically designed for fault diagnosis of planetary gear systems, achieving higher diagnostic precision and efficiency. These advances in traditional signal analysis techniques lay the groundwork for more sophisticated diagnostic methods, demonstrating the potential of integrating traditional methods with modern machine learning algorithms to improve fault diagnosis accuracy.

Despite the practicality of these traditional methods, they have inherent limitations [12]. First, they rely heavily on the expertise and experience of engineers, requiring in-depth theoretical understanding and practical skills at every stage, from data preprocessing and parameter selection to interpretation [13]. This dependence on expert knowledge means that the diagnostic process can be time-consuming and prone to human error. Moreover, the necessity of manual feature extraction and parameter tuning can introduce variability and inconsistencies in the results. Second, their effectiveness diminishes when faced with highly complex systems and high-dimensional data, which potentially leads to inaccurate analytical results. Traditional methods often struggle to handle complex nonlinear interactions in data, which may result in missed or incorrect fault diagnosis. These limitations suggest that traditional approaches may not meet the stringent standards of modern equipment fault diagnosis owing to increasing data complexity and volume. These limitations highlight the need for intelligent and robust diagnostic methods that can effectively handle large-scale data and provide accurate and reliable results with minimal human intervention.

The rapid development of deep learning has resulted in unprecedented technological advancements in the field of bearing fault diagnosis. Advanced deep learning models, such as autoencoders [14], convolutional neural networks (CNNs) [15], and residual neural networks [16], have been increasingly adopted owing to their superior ability to learn complex representations from data. These models can handle extensive datasets autonomously to identify potential fault patterns and trends, significantly reducing their reliance on expert intuition and experience. For instance, autoencoders can effectively compress and reconstruct data, highlighting deviations from normal operating patterns and making them useful for anomaly detection in bearing signals. CNNs capture spatial hierarchies in data, allowing them to automatically extract and learn features from raw vibration signals, thereby circumventing the requirements for manual feature engineering. ResNets address the vanishing gradient problem in deep networks, allowing the construction of deeper models that are capable of capturing more complex fault features. These deep learning models not only improve the accuracy and efficiency of fault diagnosis but also enable the development of more robust and scalable diagnostic systems that can adapt to various operating conditions and datasets.

For example, Wu et al. [17] employed a 1D convolutional neural network to automatically extract features from vibration signals, thereby bypassing the need for complex feature extraction techniques. This approach allows raw vibration data to be directly input into the model, facilitating end-to-end learning and reducing the likelihood of human error during feature extraction. Zhang et al. [18] developed a novel method that transforms raw signals into 2D images by using spatial representations to automatically identify fault features through a network. This transformation simplifies the traditional manual feature extraction process and enhances the ability of the mode to capture complex fault patterns. Li et al. [19] combined wavelet packet transform with CNNs, further reducing the need for manual intervention and simplifying the fault diagnosis process. This hybrid method integrates the advantages of time–frequency analysis and deep learning, providing a more comprehensive diagnostic framework. Jia et al. [20] used a shallow kernel convolutional network combined with batch normalization and the Adam optimization algorithm to improve the convergence speed of the training process and the generalization ability of their model. This combination enhances network performance by stabilizing the learning process and optimizing weight adjustments. Hao et al. [21] adopted an innovative approach by replacing the fully connected layers in traditional ResNets with global average pooling, effectively reducing model parameters and improving operational efficiency, thereby enhancing the overall performance of the system. The application of these deep learning techniques not only improves the accuracy and efficiency of fault diagnosis but also opens new avenues for optimizing early fault prediction and maintenance strategies. These advancements highlight the potential of deep learning to revolutionize the field of fault diagnosis, thereby making it more accurate, efficient, and scalable.

Despite the high effectiveness of deep learning methods in bearing fault diagnosis, there are still some limitations to their application [22]. One major issue is the insufficient consideration of feature interconnectivity, which can lead to models failing to comprehensively capture crucial feature information, thereby affecting their diagnostic performance and accuracy. Moreover, the complexity and variability of the bearing operating environment poses significant diagnostic challenges. For example, mechanical vibrations and external disturbances from other components can significantly impact data quality and reduce the effectiveness of diagnostic methods [23]. To address this issue, Chen et al. [24] proposed a method that combined a 1D denoising convolutional autoencoder (DCAE) with a 1D convolutional neural network. This method effectively removes noise from the data by training noisy input data, thereby significantly improving the accuracy of fault diagnosis. This method enhances the robustness of the diagnostic process by filtering irrelevant noise and retaining necessary fault-related features. Similarly, Xu et al. [25] developed a multi-receptive field denoising residual convolutional network that utilized a residual structure to extract deeper features, thereby enhancing the adaptability of the model to complex environmental noise.

However, previous fault diagnosis models typically employed similar network architectures, utilizing stacked convolutional layers for feature extraction followed by the direct input of the extracted features into fully connected networks. The primary method for enhancing noise resistance involves adjusting the hyperparameters (e.g., kernel size, quantity, and hidden layer size). Furthermore, existing models mainly focus on the accuracy performance in low-noise environments. In real-world scenarios, the types and intensities of noise vary significantly. Therefore, it is crucial to develop models that can handle complex noise effectively. Attention mechanisms are particularly adept at handling long-sequence data and noisy inputs, and they can adaptively capture complex dependencies in sequences, making them well suited for tasks such as time series analysis. The robustness of the model was enhanced by dynamically filtering the noise, resulting in a more accurate and reliable intelligent fault diagnosis.

To improve the diagnostic accuracy and noise resistance of bearing fault diagnosis models for rotating machinery, we propose a novel model called the MR-FuSN. This model adopts a multi-resolution attention mechanism and enhanced residual convolution to optimize the utilization of raw input data.

This paper presents a new bearing fault diagnosis model, the Multi-Resolution Fusion Selection Network(MR-FuSN). The MR-FuSN does not require complex manual feature extraction processes and demonstrates excellent robustness under noisy conditions, thereby improving the performance of fault diagnosis methods under noise interference.

The main contributions of this paper can be summarized as follows:

1. A feature extraction module is designed, combining residual convolution, multi-resolution, and attention mechanisms, effectively extracting key features at different scales and improving the detection accuracy of bearing fault diagnosis.
2. An adaptive dual-kernel channel-focusing module is developed that dynamically adjusts processing strategies based on the characteristics of the input data, thereby enhancing the model’s adaptability and diagnostic efficiency in complex data environments.
3. The model was validated using two bearing datasets and compared to other diagnostic methods, thereby demonstrating its advantages in terms of accuracy and noise resistance. These results confirm the effectiveness and potential value of the proposed model for practical applications.

## 2. Principle and Model Framework

**Figure 1:**
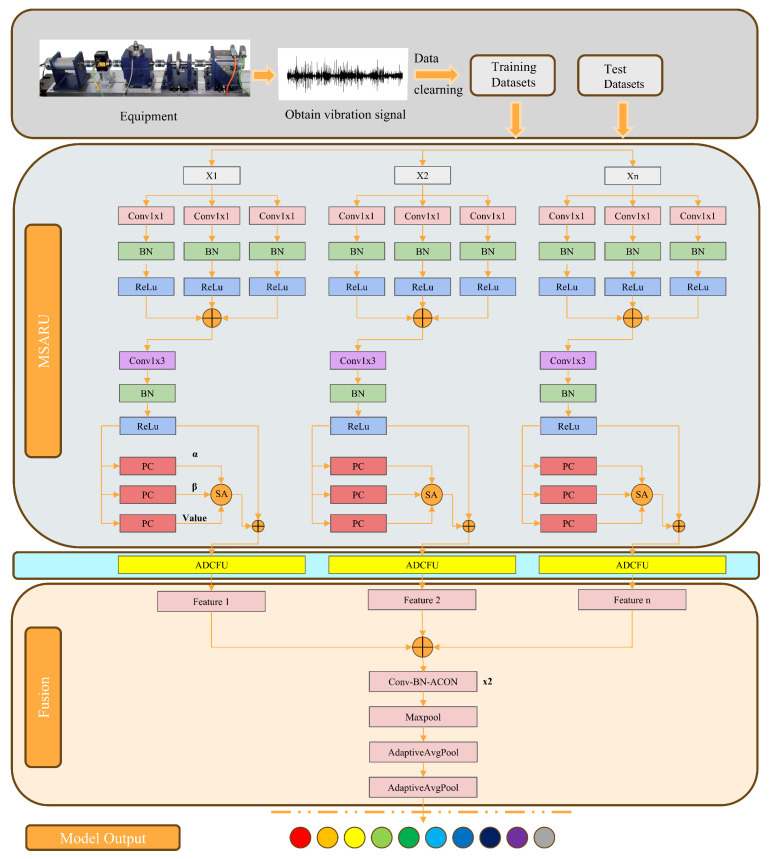
The overall architecture of MR-FuSN. SA: self-attention module; α, β, Value: query, key and value vectors for self-attention calculation; ⊕: feature fusion operation; $X_{1}$, $X_{2}$, ..., $X_{n}$: different network widths.

In this paper, a novel bearing fault diagnosis framework is proposed, as shown in Figure 1, which integrates multi-resolution processing, Multi-Level Spatial Attention Residual Unit (MSARU) and Adaptive Dual-Core Channel-Focusing Unit (ADCFU). These units not only help to identify features, but also facilitate the model to adaptively adjust its receptive field and optimize feature selection. The construction and implementation of these methods is described in detail in the following section.

### 2.1. Multi-Level Spatial Attention Residual Unit

In the field of bearing fault diagnosis, existing models based on traditional residual networks and attention mechanisms have demonstrated a certain level of effectiveness in terms of feature extraction [26]. However, their linear structure often limits their capability to process multi-scale fault signals, particularly when capturing critical low-level features [27]. These models struggle to simultaneously account for both global and local features when dealing with complex and diverse fault signals, which adversely affects overall diagnostic performance and accuracy. To address this issue and improve diagnostic accuracy in noisy environments, we propose a Multi-Scale Spatial Attention Residual Unit (MSARU) module. By combining the strengths of the residual networks and multi-scale attention mechanisms, the MSARU module effectively extracts key features at different scales, thereby significantly enhancing the performance of the model in complex fault signal scenarios.

The overall structure of the MSARU module is shown in Figure 1. Input feature *X* is first processed through a deep convolution layer [28]. The depthwise convolution, as the initial operation of the MSARU module, processes each input channel independently, significantly reducing computational complexity. Given input features with dimensions C×L, the parameter count for depthwise convolution is C×K, whereas traditional convolution requires C×C×K, resulting in a computational reduction ratio of 1/C. This design not only optimizes model efficiency but also enhances the extraction capability of critical intra-channel fault features (such as transient impacts) through local convolution kernels. Subsequently, pointwise convolution performs multi-scale fusion of these preprocessed local features across multiple parallel branches, avoiding the parameter explosion and local information loss problems that would occur with direct pointwise convolution [29]. The operation can be expressed as

(1) $$Y_{depth}^{\left(c\right)}=X^{\left(c\right)}*K_{depth}^{\left(c\right)},$$

where $Y_{depth}^{\left(c\right)}$ is the output feature of the cth channel, and $X^{\left(c\right)}$ and $K_{depth}^{\left(c\right)}$ represent the input feature and depth convolution kernel, respectively. This operation reduced the computational complexity of the model. Subsequently, the deep convolution feature $Y_{depth}$ was further processed through a 1D convolution layer combined with batch normalization (BatchNorm1d) and a ReLU activation function to improve the stability and quality of the feature representation. This process can be expressed as:

(2) $$Y_{conv}=ReLU\left(BatchNorm1d\left(Y_{depth}*K_{conv1}\right)\right).$$

Subsequently, the processed feature $Y_{conv}$ was distributed to three parallel pointwise convolution layers for multi-scale feature extraction. Each pointwise convolution layer decomposes and reconstructs the features using convolution kernels of different sizes to capture information at various scales. The operation of each pointwise convolution layer can be expressed as

(3) $$Y_{point}^{\left(i\right)}=Y_{conv}*K_{point}^{\left(i\right)},$$

where (i = 1, 2, 3) represents different scales, and $K_{point}^{\left(i\right)}$ is the corresponding pointwise convolution kernel.

After the multi-scale features are extracted through the pointwise convolution layers, they are weighted using a self-attention mechanism (SA) [30]. In the MSARU module, the different scale features $Y_{point}^{\left(1\right)}$, $Y_{point}^{\left(2\right)}$, and $Y_{point}^{\left(3\right)}$ are combined with three trainable weight parameters, α, β, and Value, respectively, which can be represented as

(4) $$\alpha =f\left(Y_{point}^{\left(1\right)}\right),\;\beta =g\left(Y_{point}^{\left(2\right)}\right),\;Value=h\left(Y_{point}^{\left(2\right)}\right)$$

Functions f(·), g(·), and h(·) are learning functions implemented through fully connected layers. These parameters can dynamically learn the relative importance of different features during the final fusion process through a self-attention mechanism, thereby achieving effective integration and precise representation of multi-scale features. The fused multi-scale feature representation is expressed as follows:

(5) $$Y_{SA}=\alpha \cdot Y_{point}^{\left(1\right)}+\beta \cdot Y_{point}^{\left(2\right)}+Value\cdot Y_{point}^{\left(3\right)}$$

Finally, the output feature $Y_{SA}$ from the self-attention mechanism is connected to the initial input feature $Y_{conv}$ through a residual connection, preserving the original information while enhancing the model’s representation capability. The residual connection formula is as follows:

(6) $$Y_{out}=Y_{conv}+Y_{SA}$$

This multi-scale feature extraction strategy not only improves the model’s ability to recognize complex fault signals but also significantly reduces the number of parameters and computational cost, lowering the risk of overfitting.

During model training, all parameters in the MSARU module, including the weight coefficients α, β, and Value, are optimized using the backpropagation algorithm. The loss function employs cross-entropy loss to evaluate the classification performance of the model:

(7) $$L=-\sum_{i=1}^{N}\left[y_{i}\log\left(p_{i}\right)+\left(1-y_{i}\right)\log\left(1-p_{i}\right)\right]$$

where *N* is the number of samples, $y_{i}$ represents the true label of the *i*-th sample, and $p_{i}$ is the model’s predicted probability. By optimizing the loss function, the model gradually learns the importance of different features in fault diagnosis, thereby improving overall diagnostic accuracy.

In summary, the MSARU module, through the organic combination of depthwise convolution, pointwise convolution, and the self-attention mechanism, can effectively extract and integrate various feature information in complex multi-scale fault signal environments, significantly enhancing the model’s diagnostic performance and robustness.

### 2.2. Adaptive Dual-Core Channel-Focusing Unit

**Figure 2:**
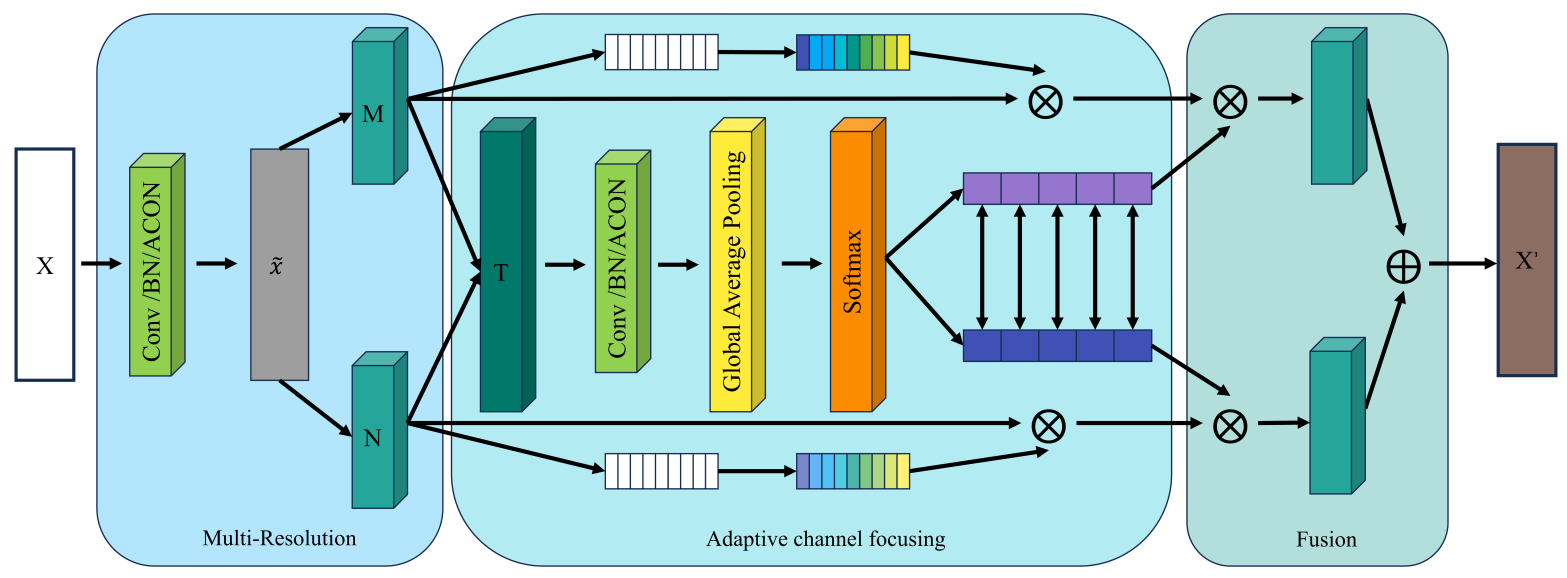
Adaptive Dual-Core Channel-Focusing Unit. ⊗: element-wise multiplication; ⊕: feature fusion.

To enhance the model’s sensitivity to domain-invariant features and adaptively adjust the receptive field range, this section proposes an Adaptive Dual-Kernel Channel-Focusing Unit (ADCFU) based on channel attention. As shown in Figure 2, this module dynamically adjusts processing strategies according to the characteristics of the input data, strengthens the representation of domain-invariant features, and effectively suppresses irrelevant or redundant information, thereby improving the model’s adaptability and diagnostic efficiency in complex data environments [31].

The ADCFU module consists of three main components: multi-resolution feature extraction, multi-channel adaptive focusing, and feature fusion. First, the input feature *X* undergoes convolution, normalization, and activation operations to generate the initial feature $\tilde{X}$, which is then processed using two convolutional kernels of different sizes. Specifically, $M=F_{5}\left(\tilde{X}\right)$ with a kernel size of 5 was used to capture short-range features, whereas $N=F_{9}\left(\tilde{X}\right)$ with a kernel size of 9 was chosen to integrate long-range features. This dual-kernel design enables the model to understand and represent input data from both local and global perspectives, thereby capturing multi-scale information more effectively. Smaller kernels help detect short-term variations and fine details, while larger kernels capture broader patterns and trends, providing a comprehensive understanding of the multi-level features in the input data [32].

After obtaining the multi-resolution features *M* and *N*, a channel attention [33] module is applied for adaptive channel focusing. First, the two features *M* and *N* are summed and passed through a shared convolutional layer T=Conv(M+N) to extract the initial fused features. Subsequently, global average pooling (GAP) is applied to *T* to generate the global feature representation GAP(T). The pooled feature representation is passed through a fully connected layer and a Softmax activation function to generate the attention weights for each channel:

(8) $$CAM\left(M,N\right)=\sigma \left(W_{2}\cdot ReLU\left(W_{1}\cdot GAP\left(T\right)\right)\right),$$

where $W_{1}$ and $W_{2}$ are trainable weight matrices for learning inter-channel dependencies, and σ(·) denotes the Sigmoid function that compresses attention weights to [0,1], dynamically enhancing key channels while suppressing noise-related features. This attention mechanism automatically adjusts the weights of different channels based on the context information of the input data, highlighting critical features with diagnostic value while suppressing interference from redundant information [34]. The generated weights were then applied to *M* and *N* to obtain the following adaptively adjusted feature representations:

(9) $$M^{\prime }=CAM\left(M,N\right)\cdot M,\;N^{\prime }=CAM\left(M,N\right)\cdot N$$

After feature focusing, the adjusted features $M^{\prime }$ and $N^{\prime }$ are further fused to generate the final output features. This fusion process is achieved by adding attention-adjusted features to the initial multi-resolution features as follows:

(10) $$X^{\prime }=M\cdot M^{\prime }+N\cdot N^{\prime }$$

This fusion strategy not only ensures the effective combination of multi-scale information but also enhances the precision of feature representation through the channel attention mechanism, thereby improving the model’s diagnostic capability and robustness in complex environments.

With this design, the ADCFU module can adaptively adjust the receptive field range and flexibly control the importance of different channel features, thereby enabling the model to exhibit stronger feature extraction capabilities and higher diagnostic efficiency when dealing with complex and variable fault signals.

### 2.3. Multi-Resolution Fusion Strategy

The fusion of multi-resolution features is a critical aspect of bearing fault diagnosis [35]. Compared to single-resolution features, multi-resolution features provide a more comprehensive and representative insight into the model. By integrating features at various resolutions, the model can more effectively identify fault signals of different frequencies, thereby enhancing diagnostic accuracy and robustness.

First, the fusion of multi-resolution features significantly improves the model’s capability to recognize fault signals at different frequencies [36]. At lower resolution levels, the model can capture the low-frequency components of the fault signals, which are typically associated with initial symptoms or subtle changes in the system. At higher resolution levels, the model can detect high-frequency components that often reflect the severity of faults or abrupt changes. By considering both low-frequency and high-frequency features simultaneously, the model provides a more comprehensive understanding of the fault signals, significantly improving the fault identification accuracy and sensitivity.

Second, multi-resolution feature fusion enhances the model’s robustness and generalization capability. In complex industrial environments, the fault signals are often affected by noise and interference. Combining features of different resolutions can effectively reduce the impact of noise and improve the model’s ability to capture essential features. For example, low-resolution features help the model ignore some high-frequency noise, whereas high-resolution features emphasize subtle changes in the fault signals [37]. This fusion strategy of multi-resolution features enhances the model’s ability to represent features across multiple scales. Given that bearing fault signals inherently possess multi-scale characteristics, where different scales reveal different aspects of faults, the model can capture a complete view of the fault signals at various scales, thereby improving its ability to identify and locate the fault source.

In the model’s multi-resolution feature fusion component, features at different resolutions are processed through separate channels. Initially, the features are processed at each resolution level and fused using a weighted aggregation mechanism. Specifically, let the low-resolution, medium-resolution, and high-resolution features be represented as ${\mathrm{feature}}_{1}$, ${\mathrm{feature}}_{2}$, and ${\mathrm{feature}}_{3}$, respectively. The fusion process can be expressed as

(11) $$F={Ag}_{1}\left({feature}_{1}\right)⊕{Ag}_{2}\left({feature}_{2}\right)⊕\ldots ⊕{Ag}_{n}\left({feature}_{n}\right),$$

where ${Ag}_{1},{Ag}_{2},\ldots ,{Ag}_{n}$ represent the weight matching functions for different features, and ⊕ denotes the feature concatenation operation. Through this weighted aggregation process, the model can capture and integrate the fault information from different resolutions at various scales.

Fused feature *F* is then fed into the classifier module, which consists of a series of convolutional and fully connected layers. First, the fused features are processed through two convolutional operations (Conv-BN-ACON), which further extract features and compress the data dimensions during the convolution process. The convolution operation is expressed as

(12) $$Y=ACON\left(BN\left(Conv(F)\right)\right)$$

where Conv denotes the convolution operation, BN represents the batch normalization layer, and ACON is the activation function. The features processed through convolution were further extracted and dimensionally reduced using Maxpool and AdaptiveAvgPool layers, preparing the features for subsequent classification.

The pooled features are then fed into fully connected layers, where a multi-layer perceptron (MLP) learns the high-level representation of fault signals. The final classification layer maps the features to specific fault categories and outputs the corresponding classification results, which can be expressed as

(13) $$\hat{y}=Softmax\left(W\cdot Y+b\right)$$

where *W* is the weight matrix of the fully connected layer, *b* is the bias term, *Y* is the pooled feature representation, and Softmax is used to normalize the output into a probability distribution. The final classification output includes multiple class labels corresponding to different fault types.

By effectively combining multi-resolution feature fusion and the classifier module, the model can simultaneously capture feature information at various scales and accurately identify fault types in a high-dimensional feature space. This multi-resolution feature fusion strategy significantly improves the fault identification accuracy and robustness of the model, especially in complex industrial environments and noise interference, demonstrating stronger adaptability and generalization capability.

In conclusion, the model structure combines multi-resolution feature fusion, and the classifier module enables comprehensive capture of diverse characteristics of bearing fault signals across different scales, providing an efficient and reliable solution for bearing fault diagnosis.

## 3. Fault Diagnosis Process Based on the MR-FuSN Model

The fault diagnosis process based on the MR-FuSN model is shown in Figure 1 and mainly includes data preprocessing, model training and prediction, and result analysis. First, the vibration signals under different working conditions were collected through the experimental platform, and the raw data were cleaned and processed to ensure the integrity and validity of the data. Next, the vibration signals were divided into samples of fixed lengths to construct the datasets, which were then divided into training and testing sets. In the model training and prediction stages, the training set was used to train the model until its accuracy reached the expected standard. Subsequently, the test set was input into the trained model for prediction to evaluate its performance on unknown data. Finally, the prediction results were analyzed in detail, and the model performance was quantitatively evaluated by calculating the accuracy, standard deviation, and other metrics to verify the effectiveness and robustness of the model in fault diagnosis tasks.

## 4. Experimental Verification

To evaluate the performance of the MR-FuSN model, we conducted experiments on two distinct datasets and assessed the classification accuracy of four comparative methods through benchmark testing. Hyperparameter optimization via grid search determined the configuration: a batch size of 32 combined with a learning rate of 0.001, strategically chosen to balance training efficiency and model convergence. The training duration was ultimately fixed at 200 epochs based on stabilization patterns observed in the validation loss curve. Adam optimizer was adopted as the core training algorithm.

### 4.1. Description of Experimental Datasets

**Figure 3:**
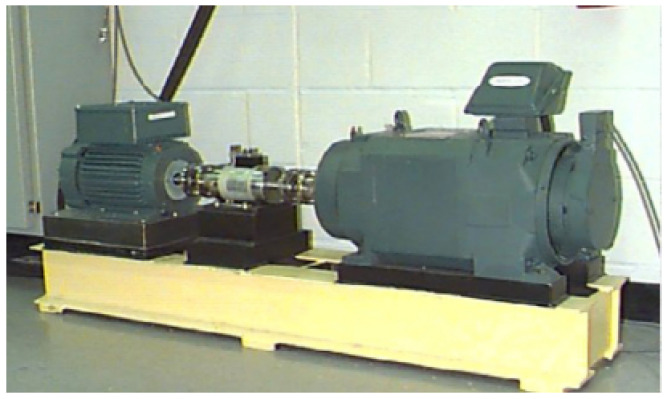
The test rig of $D_{1}$.

Dataset $D_{1}$ was sourced from the Case Western Reserve University Bearing Data Center and included various bearing fault modes. The experimental setup is shown in Figure 3. In this setup, bearing defects were artificially induced using an electrical discharge machining process, creating three defect sizes with diameters of 7, 14, and 21 mils. Vibration data were collected using an accelerometer mounted on the housing with a magnetic base. These vibration signals were sampled at a rate of 12,000 samples to analyze the failures of the drive-end bearing. Table 1 presents the raw vibration signals under ten different operating conditions. Each class contains 400 samples (1-s segments). Each fault type and severity combination contains 400 samples, where each sample corresponds to a 1-s signal segment (12,000 data points). The dataset was split into training and testing sets with an 7:3 ratio (280 training samples and 120 testing samples per class), ensuring stratified sampling to maintain class distribution consistency.

**Figure 4:**
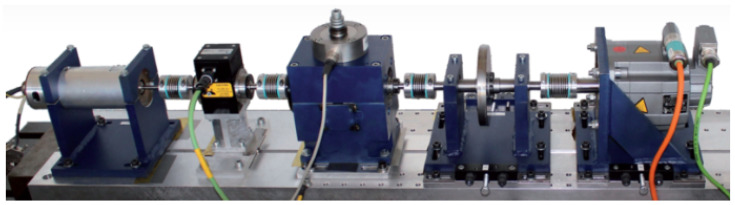
The test rig of $D_{2}$.

The bearing dataset $D_{2}$ was sourced from Paderborn University, Germany. As shown in Figure 4, the test rig consists of the following components: an electric motor, a torque measurement shaft, bearing test unit, flywheel, and load motor. The data were sampled at rates up to 64 kHz. The dataset includes 32 types of faulty bearings, comprising 6 healthy bearings, 14 naturally accelerated damaged bearings, and 12 artificially damaged bearings. The $D_{2}$ subset used in this paper primarily consists of ten states, including one healthy bearing, five naturally accelerated damaged bearings, and four artificially damaged bearings, as shown in Table 2. The training–test split followed a 7:3 ratio (280 training and 120 testing samples per class), with temporal continuity preserved to avoid overlapping segments between sets.

### 4.2. Accuracy Comparison with Other Methods

In the experimental section, ResNet06, TDSMAE, WDCNN [38], and GRU-WDCNN [39] were selected as baseline models for comparison. This selection was based on three research considerations: First, ResNet06, as a classic residual network, establishes a performance benchmark for spatial feature extraction; TDSMAE achieves multi-scale feature enhancement through variable-width convolution kernels, representing the latest advances in subtle feature extraction; WDCNN employs wide-kernel convolution and stride optimization strategies, demonstrating advantages in feature capture under strong noise environments; GRU-WDCNN extends temporal modeling capabilities through gated recurrent units. These four models correspond to four major technical directions in fault diagnosis: spatial feature learning, multi-scale analysis, noise-resistant architecture, and temporal dependency modeling. Second, regarding the core challenges specific to bearing fault diagnosis, including weak impact signal detection, operational noise interference, and vibration signal temporal correlation, the selected models have demonstrated superiority in specific scenarios in their original literature, constituting a comprehensive performance evaluation benchmark.

In the experimental setup, the step size was set to 64, and the sample shape was 2048 × 1. The training process used the Adam optimization algorithm for gradient updates, and the hyperparameters were optimized through grid search. The model was trained for a total of 200 epochs with a batch size of 32 and an initial learning rate of 0.001. All experiments were conducted on a computer equipped with an Intel Core i7-12700K processor (Intel, Santa Clara, CA, USA) and an NVIDIA GeForce RTX 3090 GPU (NVIDIA, Santa Clara, CA, USA) using the PyTorch (v2.4.0) deep learning framework [40] for model construction and experimental simulation.

A five-fold cross-validation was performed for each method, and the final results were averaged over the five experiments, which was done to mitigate the local optimal error that may occur in a single experiment. The results are presented in Table 3. The effectiveness and reliability of the models were evaluated using “accuracy” and “standard deviation” as metrics. For the $D_{1}$ training set, all the models achieved 100% accuracy during the five training runs. On the D1 test set, MR-FuSN achieved the best performance, with an accuracy of 99.98% and a standard deviation of 0.02. The accuracy of the other four methods ranged from 99.91% to 99.96%, with standard deviations between 0.03% and 0.06%, which were slightly lower than that of MR-FuSN, with a difference in accuracy of approximately 0.03%. Because the $D_{1}$ dataset exhibited significant differences between categories, all methods performed well during testing.

The MR-FuSN also demonstrated excellent performance during the training and testing phases in the $D_{2}$ dataset. As shown in Table 3, the accuracy of the training set was 99.98%, with a standard deviation of 0.19, and the accuracy of the test set was 99.92%, with a standard deviation of 0.09. Compared to the other models, the MR-FuSN shows greater flexibility and robustness in handling complex features owing to the functionality of the ADCFU module, which adaptively adjusts feature extraction, enhances the representation of invariant features, and suppresses irrelevant information. Consequently, the MR-FuSN maintained a strong performance even on the $D_{2}$ dataset.

**Figure 5:**
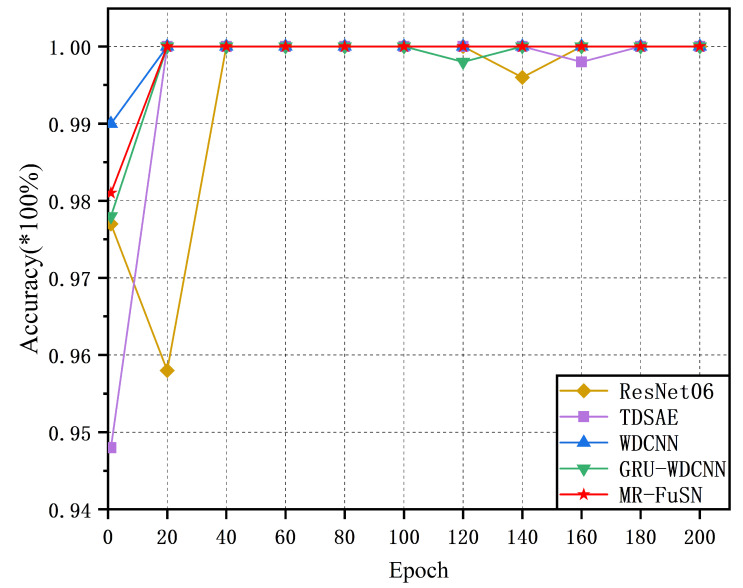
The convergence curve of $D_{1}$.

**Figure 6:**
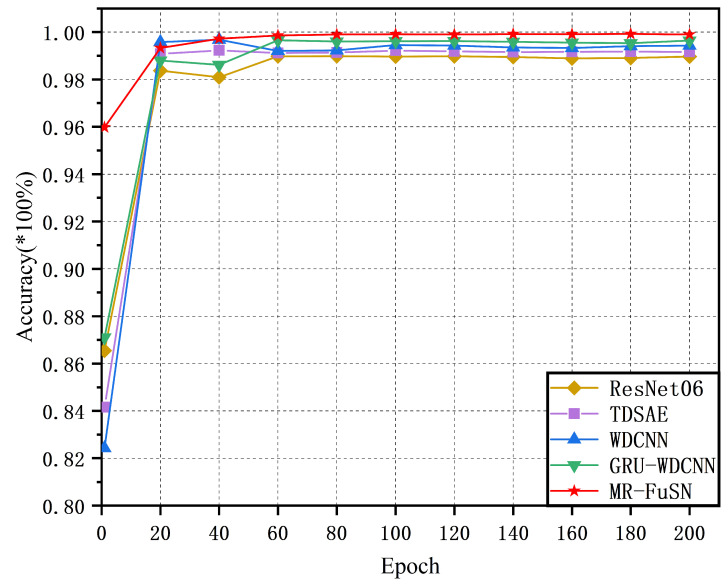
The convergence curve of $D_{2}$.

The convergence curves of the five methods based on the best models selected from the five-fold cross-validation are shown in Figure 5 and Figure 6. During training, the training accuracy of each epoch was calculated as the average accuracy of all batches. As shown in Figure 5, the five methods converged relatively quickly on the $D_{1}$ dataset. Moreover, the MR-FuSN exhibited the smallest fluctuation in its convergence curve compared whit the other models, indicating that the MSARU and ADCFU modules contributed to more stable gradient updates. As shown in Figure 6, the performance of the five methods on the other datasets showed a similar trend. The MR-FuSN maintained the fastest convergence speed and after reaching convergence and sustained a high level of accuracy with minimal fluctuations, demonstrating optimal performance. This indicates that the MR-FuSN can achieve a stable state more quickly while maintaining a high performance when dealing with complex data.

### 4.3. Noise Interference Experiment

Vibration signals in real industrial environments are frequently contaminated by complex noise, which poses significant challenges to fault feature extraction and diagnosis. Noise not only obscures subtle characteristics of fault signals (such as periodic impulses and harmonic components) but can also lead to feature confusion (e.g., spectral overlap between high-frequency noise and fault-induced impulses), thereby substantially degrading the diagnostic accuracy and robustness of models. Consequently, research on bearing fault diagnosis methods under noisy conditions holds crucial theoretical significance and practical application value.

To simulate the effects of noise on fault signals under actual operating conditions, this experiment introduced Gaussian white noise to contaminate original vibration signals. This approach enables systematic investigation of how different noise intensities affect model performance while validating the robustness of the proposed method in noisy environments. Through this methodology, we can more authentically assess a model’s fault diagnosis capabilities under noise interference. A lower SNR (signal-to-noise ratio) indicates a higher level of noise contamination. The SNR is defined as

(14) $$\mathrm{SNR}=10\times \log_{10}\frac{E_{S}}{E_{n}}$$

where $E_{S}$ and $E_{n}$ represent the signal and noise powers, respectively.

To validate the superiority of the MR-FuSN in noisy environments, we compared its performance with that of ResNet06, TDSMAE, WDCNN, and GRU-WDCNN under the same experimental settings as described above. The results are presented in Table 4. On noisy datasets $D_{1}$ and $D_{2}$, the MR-FuSN demonstrated the highest diagnostic accuracy across all SNR levels. At SNR = 0 dB, the MR-FuSN achieved 99.97% accuracy on $D_{1}$ and 99.85% on $D_{2}$, surpassing WDCNN (90.58% and 88.45%) and GRU-WDCNN (92.33% and 90.12%). The experiments demonstrate that wide convolutional kernels and attention mechanisms are effective for extracting useful fault features, even from noisy data. For the $D_{1}$ dataset, the MR-FuSN, WDCNN, and GRU-WDCNN generally outperformed ResNet06 in terms of accuracy, proving that wide convolutional kernels have a significant advantage in extracting fault features in noisy environments. Their primary function is to capture a broader range of feature information, allowing them to effectively retain key features in the signal, even under complex noise backgrounds. Compared to narrow convolutional kernels, wide convolutional kernels cover a larger receptive field, enhancing the ability of the model to perceive low-frequency fault signals and global features. This characteristic enables the model to extract stable fault features despite noise interference, thereby improving noise resistance and diagnostic accuracy. Compared to ResNet06, WDCNN, and GRU-WDCNN, the MR-FuSN achieved higher accuracy owing to the combination of the ADCFU and MSARU modules. Unlike traditional CNNs, ResNets, and attention mechanisms, the MR-FuSN is more flexible in capturing long-term fault information and dealing with noise. The ADCFU module adaptively adjusts the receptive field to extract features at different scales, effectively filtering out noise and enhancing the expression of the key features. The MSARU module further refines the localization of fault features through multi-scale attention mechanisms, allowing the MR-FuSN to maintain a high level of diagnostic capability and robustness even in complex environments.

### 4.4. Ablation Experiment

To verify the effectiveness of each module within the MR-FuSN and the appropriateness of parameter selection, an analysis was conducted under a signal-to-noise ratio of 0 dB. Based on datasets $D_{1}$ and $D_{2}$, the fault diagnosis accuracy of the MR-FuSN under different ablation scenarios was investigated.

#### 4.4.1. Comparison of Different Network Architectures

To evaluate the performance of the MDARU, ADCFU, and multi-resolution fusion strategy within the model, this section employed an experimental design based on three different network architectures. These designs aimed to validate the effectiveness of each key component within the overall framework independently.

Scheme A: To investigate the role of MDARU in fault diagnosis, a modified version of the model was designed with the MDARU unit removed, while the rest of the model remained unchanged.

Scheme B: To assess the impact of the ADCFU module on fault diagnosis performance, the ADCFU part was removed, keeping the rest of the model intact.

Scheme C: To evaluate the effectiveness of the multi-resolution fusion strategy, a simplified model was designed with feature fusion performed by concatenation along the channel dimension, while other components remained unchanged.

**Figure 7:**
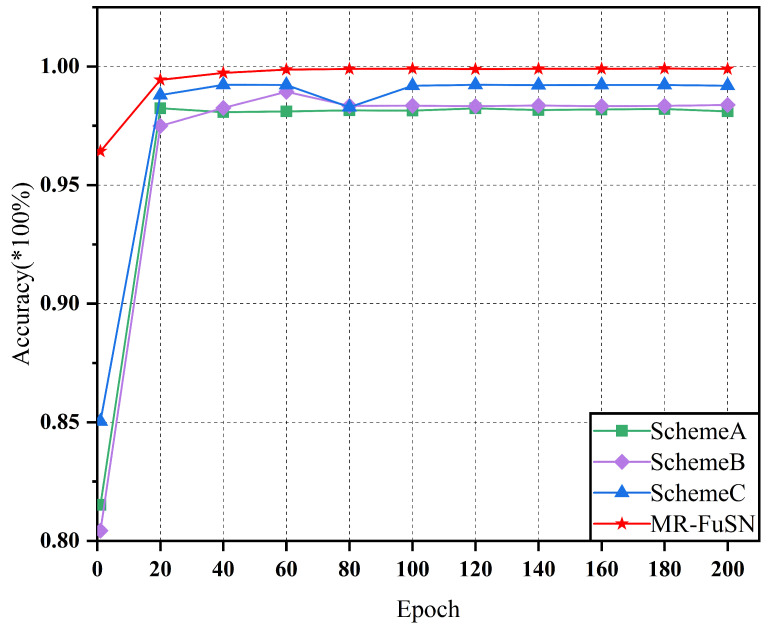
Accuracy of different network architectures under dataset $D_{1}$.

**Figure 8:**
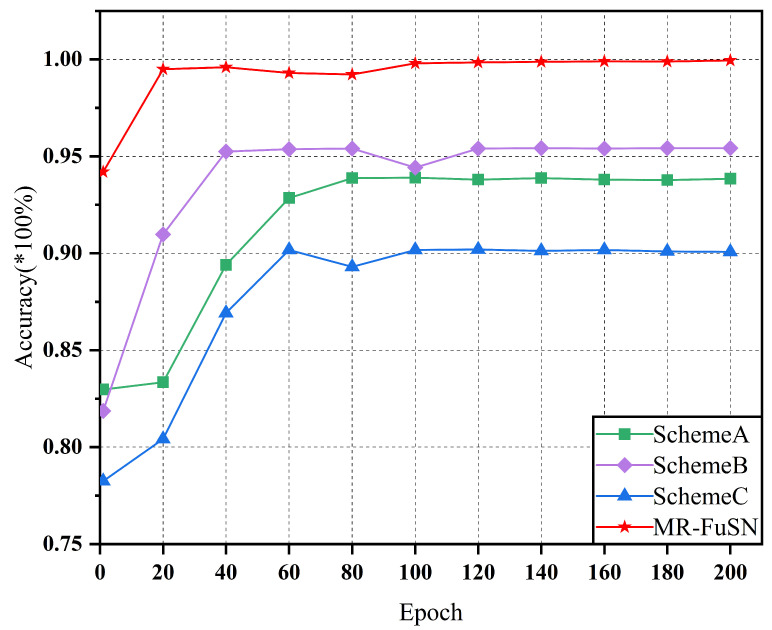
Accuracy of different network architectures under dataset $D_{2}$.

Through these three experimental schemes, we not only independently evaluated the contribution of each module to the overall fault diagnosis performance but also further validated the comprehensive advantages of the MR-FuSN in bearing fault diagnosis. As shown in Figure 7 and Figure 8, in Scheme A, after removing the MDARU module, the diagnostic accuracy for both datasets decreased. This indicates the critical role of the MDARU in feature extraction and accuracy improvement for fault diagnosis. The MDARU module effectively identifies and enhances the global dependencies between the input features through a self-attention mechanism. This mechanism not only helps the model distinguish and emphasize features crucial in fault diagnosis, but also suppresses irrelevant noise features, thereby optimizing the feature extraction process. In Scheme B, after removing the ADCFU module, the accuracy of the model declined, demonstrating the contribution of this part of the model to fault diagnosis. The ADCFU module combines the two convolutional kernels of different sizes, allowing the model to extract various local details while effectively suppressing noise. This dual-kernel design ensures high accuracy in noisy environments, especially under complex and variable operating conditions. In Scheme C, using a simplified fusion strategy resulted in a significant decline in accuracy compared to the complete MR-FuSN model, clearly illustrating the importance of the multi-resolution fusion strategy in capturing multi-scale information from fault signals. The multi-resolution feature extraction module effectively captures multi-scale information of fault signals by utilizing feature maps of different resolutions, thereby improving diagnostic accuracy. The ablation experiment results show that removing this module leads to a noticeable drop in accuracy, further proving the superior performance of the MR-FuSN.

In summary, the MDARU, ADCFU, and multi-resolution fusion strategies in the MR-FuSN significantly enhance the model’s stability and accuracy, as well as play a crucial role in bearing fault diagnosis tasks under complex noise backgrounds and various fault scenarios.

#### 4.4.2. Network Width Parameter Selection

This section aims to validate the appropriateness of the multi-resolution parameter selection in the MR-FuSN network. A series of comprehensive experiments were conducted in the context of bearing fault diagnosis to verify the multi-resolution capability of the MR-FuSN network. The results indicate that optimizing the network resolution parameters can improve model performance. Selecting an appropriate network multi-resolution is crucial for bearing fault diagnosis, as it directly impacts the model’s ability to identify fault-related features. In our experiments, we used four different network widths: 4, 6, 8, and 10.

**Figure 9:**
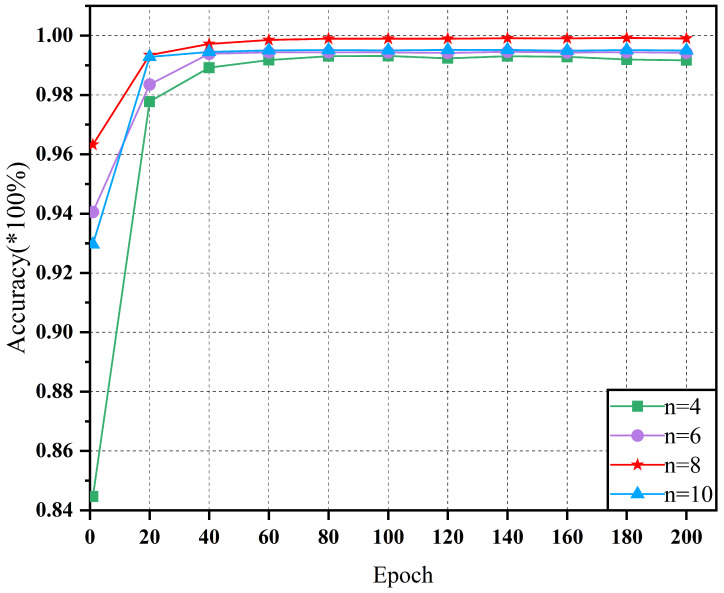
Fault diagnosis accuracy for different network parameters under dataset $D_{1}$.

Figure 9 shows the variation in the model accuracy for different network widths on the $D_{1}$ dataset. As illustrated, when the network width increased from 4 to 8, the diagnostic accuracy of the model improved significantly. For example, when the network width was n=4, the model accuracy initially increased rapidly but then stabilized, ultimately reaching approximately 96.12%. This suggests that the basic network structure can effectively learn fundamental fault patterns but struggles to capture complex features. When the network width was n=6, the model accuracy gradually increased during training and eventually stabilized at approximately 96.25%. Compared to n=4, the network width of n=6 demonstrates better performance in capturing complex features. At n=8, the model achieved the highest accuracy of approximately 99.98%. This indicates that with this parameter setting, the model attains an optimal balance between the feature extraction and generalization capability. However, when the network width was further increased to n=10, the model accuracy initially increased more rapidly in the early training stages, but then stabilized at approximately 94%. This suggests that an excessively wide network may lead to overfitting, resulting in reduced generalization performance on the test set, despite improved performance on the training set.

**Figure 10:**
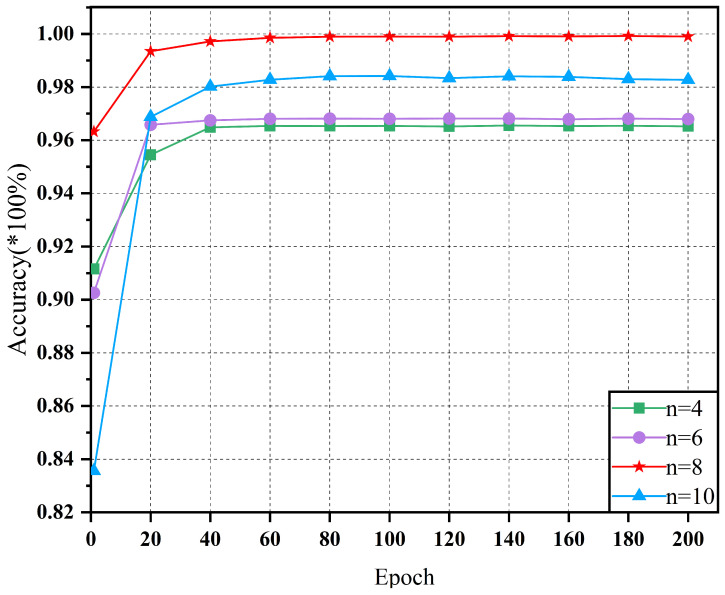
Fault diagnosis accuracy for different network parameters under dataset $D_{2}$.

Figure 10 presents the accuracy trends of the model with different network widths on the $D_{2}$ dataset, which shows a similar overall pattern to the results on the $D_{1}$ dataset. As the network width increased, the diagnostic accuracy of the model initially increased and then decreased, indicating that an appropriate network width can enhance the feature extraction capability and diagnostic accuracy. However, an excessiverly large network width may cause the model to overfit, thereby impairing its generalization capability. At n=8, the model again demonstrated the best diagnostic performance on the $D_{2}$ dataset, further confirming the effectiveness of this network structure parameter in balancing feature extraction and model complexity.

In summary, the network width significantly influences the fault diagnosis performance of the model. For both datasets $D_{1}$ and $D_{2}$, as the network width increased from 4 to 8, the diagnostic accuracy of the model improved noticeably, suggesting that a wider network structure can better capture complex features. However, when the network width is increased further to 10, the model performance exhibited a downward trend, primarily owing to overfitting, which reduces the generalization capability. Therefore, a moderate network width achieves an ideal balance between feature extraction and model complexity, effectively enhancing the diagnostic accuracy and robustness of the model under various noise environments. By carefully selecting the network width, it is possible to maintain efficient feature extraction capabilities while avoiding overfitting.

## 5. Conclusions

This paper proposes a novel fault diagnosis model, the MR-FuSN, consisting of two modules: the ADCFU and MSARU. The ADCFU module adaptively extracts multi-scale fault features from input data to effectively suppress noise interference. The MSARU module integrates multi-resolution analysis, residual convolution, and attention mechanisms, enhancing the model’s flexibility and robustness when processing complex signals. Experimental results demonstrate that MR-FuSN significantly outperformed other baseline models in diagnostic accuracy across two datasets, particularly exhibiting superior anti-interference capability under noisy conditions. Overall, the MR-FuSN design combining the ADCFU and MSARU modules exhibited significant advantages in fault feature extraction and model generalization capability. However, the model still has applicability limitations: the MR-FuSN relies on annotated data, while industrial scenarios typically suffer from scarce fault samples and high annotation costs. Additionally, the model’s performance under variable rotational speeds or sudden load change conditions remains unverified. Future work will focus on time–frequency joint feature enhancement and dynamic operating condition adaptation strategies to facilitate practical applications of this model in complex industrial scenarios.

## Figures and Tables

**Table 1:** Description of datasets $D_{1}$.

Class Label	Fault Diameters	Number of Samples	Fault Types
0	0	400	Normal
1	0.007	400	IF
2	0.007	400	BF
3	0.007	400	OF
4	0.014	400	IF
5	0.014	400	BF
6	0.014	400	OF
7	0.021	400	IF
8	0.021	400	BF
9	0.021	400	OF

**Table 2:** Description of dataset $D_{2}$.

Class Label	Bearing Code	Number of Samples	Fault Types
0	K003	400	Normal
1	KA04	400	OR
2	KA16	400	OR
3	KI04	400	IR
4	KI16	400	IR
5	KI18	400	IR
6	KA01	400	OR
7	KA05	400	OR
8	KI01	400	IR
9	KI05	400	IR

**Table 3:** Mean precision and standard deviation on different datasets.

Model	$D_{1}$ (Train)	$D_{1}$ (Test)	$D_{2}$ (Train)	$D_{2}$ (Test)
MR-FuSN	100.00±0.01%	99.98±0.02%	99.98±0.19%	99.92±0.09%
ResNet	100.00±0.03%	99.91±0.05%	99.83±0.31%	99.75±0.26%
TDSAE	100.00±0.01%	99.93±0.03%	99.89±0.26%	99.65±0.21%
WDCNN	100.00±0.02%	99.95±0.04%	99.91±0.13%	99.77±0.15%
GRU-WDCNN	100.00±0.01%	99.96±0.06%	99.93±0.21%	99.80±0.19%

**Table 4:** Diagnostic accuracy of different models on noisy datasets $D_{1}$ and $D_{2}$.

Model	SNR = −5 dB	SNR = 0 dB	SNR = 10 dB	SNR = −5 dB	SNR = 0 dB	SNR = 10 dB
	($D_{1}$)	($D_{1}$)	($D_{1}$)	($D_{2}$)	($D_{2}$)	($D_{2}$)
MR-FuSN	99.33%	99.97%	100.00%	98.45%	99.85%	99.98%
	±1.24%	±0.12%	±0.01%	±1.18%	±0.15%	±0.02%
ResNet	49.49%	66.75%	99.10%	48.12%	62.34%	97.89%
	±7.52%	±5.25%	±2.00%	±6.33%	±4.78%	±1.45%
TDSMAE	50.36%	68.77%	99.47%	52.11%	65.23%	98.56%
	±3.91%	±1.67%	±0.55%	±4.02%	±2.89%	±0.67%
WDCNN	80.71%	90.58%	99.81%	78.92%	88.45%	99.45%
	±3.42%	±0.32%	±0.27%	±3.15%	±1.12%	±0.25%
GRU-	87.90%	92.33%	100.00%	85.67%	90.12%	99.92%
WDCNN	±1.62%	±0.42%	±0.02%	±2.01%	±0.98%	±0.03%

## Data Availability

The authors do not have permission to share the data.
